# Switching to a Dual-Drug Regimen in HIV-Infected Patients Could Be Associated With Macrophage Activation?

**DOI:** 10.3389/fmed.2021.712880

**Published:** 2021-08-12

**Authors:** Matteo Vassallo, Jacques Durant, Roxane Fabre, Michel Ticchioni, Laurene Lotte, Audrey Sindt, Annick Puchois, Anne De Monte, Renaud Cezar, Pierre Corbeau, Christian Pradier

**Affiliations:** ^1^Department of Internal Medicine/Infectious Diseases, Cannes General Hospital, Cannes, France; ^2^Unité de Recherche Clinique Cote d'Azur (UR2CA), Centre Hospitalier Universitaire Pasteur 2, Nice, France; ^3^Infectious Diseases Department, Université Côte d'Azur, Nice, France; ^4^Public Health Department, Nice University Hospital, Côte d'Azur University, Nice, France; ^5^CoBTeK Lab, Nice University Hospital, Centre Memoire de Ressource et de Recherche, Côte d'Azur University, Nice, France; ^6^Laboratory of Immunology, Nice University Hospital, Université Côte d'Azur, Nice, France; ^7^Multipurpose Laboratory, Cannes General Hospital, Cannes, France; ^8^Laboratory of Virology, Nice University Hospital, Université Côte d'Azur, Nice, France; ^9^Laboratory of Immunology, Nimes University Hospital, Nimes, France

**Keywords:** HIV, succesful treatment, simplification strategies, inflammation, macrophage activation

## Abstract

**Objectives:** The aim of this study was to evaluate the effect on immune activation of switching from a triple-drug to a dual-drug regimen in HIV-1 infected patients on successful combination antiretroviral treatment (cART). Immunadapt is a prospective study evaluating the impact of cART simplification on immune activation.

**Methods:** We prospectively collected blood samples in HIV-1 infected patients on stable and successful cART switching from triple to dual regimens as a simplifying strategy. We compared immune activation markers: high sensitivity CRP, IL-1, IL-6, IL-8, IP-10, MCP-1, TNF-alpha, soluble CD14 (sCD14), soluble CD163 (sCD163), lipopolysaccharide binding protein, and D-dimer before cART change and at least 6 months after the switch. Patients were stratified according to low or high risk factors of immune activation (low CD4 nadir, previous AIDS-defining condition or very-low-level viremia during follow-up).

**Results:** From April 2019 to May 2020, 20 subjects were included (mean age 57 years, 25 years since HIV infection, CD4 666 cells/mm3, CD8 766 cells/mm3, CD4/CD8 0.94, CD4 nadir 326 cells/mm3, 15% with AIDS, 18 years on cART, 6 cART regimens received, current cART duration: 56 months). Fourteen patients were prescribed Dolutegravir + Rilpivirine and six received Dolutegravir + Lamivudine. After 6.9 months, a significant sCD163 increase (+ 25.5% vs. + 0.5%, *p* = 0.02) was observed in subjects with high risk factors, despite maintaining a viral load <50 cp/ml.

**Conclusion:** cART simplification in favor of dual therapy is associated with macrophage activation in patients at risk of immune activation despite sustained virological control. Risk factors should thus be considered before generalizing such strategies.

## Introduction

Triple-drug antiretroviral regimens are the standard of care for controlling HIV viral replication. Thanks to such strategies, life expectancy has dramatically increased in HIV+ patients (1).

These regimens typically include two nucleoside reverse transcriptase inhibitors (NRTI), associated with one of the following drug classes: a non-nucleoside reverse transcriptase inhibitor (NNRTI), an integrase strand transfer inhibitor (INSTI) or a protease inhibitor (PI) (2, 3).

However, the increasing prevalence of aging-related comorbidities, i.e., cardiovascular, renal and metabolic disorders, (4), requires risk-reduction strategies in view of potential adverse effects of long-term combination antiretroviral therapies (cART) (5, 6).

In successfully treated patients, simplification of cART in favor of certain dual-drug regimens has been shown to maintain virological control, with potential reduction of long-term adverse effects (7, 8). Several dual drug regimens have thus been introduced, generally with a NRTI-sparing cART or with a single NRTI associated with another HIV drug class.

Even in naïve patients, dual therapy with Lamivudine, a NRTI, and Dolutegravir, an INSTI, has been approved and proven non-inferior in terms of virological control in plasma and semen when compared with standard triple-drug regimens (9).

However, as triple-drug regimens have the potential to achieve adequate penetration in tissue and cellular reservoirs, concerns have been raised about the effects of dual therapies on immune activation, which is a well-known predictor of clinical events and mortality in HIV-infected patients (10). In particular, a low CD4 nadir, previous AIDS-defining conditions and residual viremia have been identified as risk factors for immune activation (11, 12).

Few studies have investigated markers of immune activation in patients simplifying their treatment (13, 14).

Our aim was to measure trends in immune activation markers according to risk factors for immune activation in patients simplifying their treatment from a 3-drug- to a 2-drug regimen.

## Methods

### Study Design and Participants

Immunadapt is a single-arm prospective study, aiming to assess the impact on immune activation markers of switching from a triple-drug to a dual-drug therapy in people living with HIV on stable cART. Patients were selected among those followed in the Department of Internal Medicine in Cannes General Hospital and the Infectious Diseases Department in Nice University Hospital, France. Each individual routinely followed in our Departments and filling the inclusion criteria received a proposal to participate to this study. Therefore, the decision of the antiretrovirals prescribed did not depend from the researchers of the study, but only from the physician who took in care each subject for its routine follow-up. Recruitments were continued until attempting the goal of 20 inclusions, which was the estimated sample size necessary for the analysis.

Inclusion criteria were HIV-1 infected subjects on stable and successful cART (viral load <50 copies/ml for at least 6 months, measured with Xpert^©^ viral load or Aptima HIV Quant Dx, Hologic), switching from a triple-drug to a dual-drug regimen as a simplification strategy.

Exclusion criteria were subjects who were not on stable and successful cART, or those whose treatment included a different number of compounds, such as those switching from a quadruple to a triple-drug regimen.

This study was approved by the Paris Ethics Committee (Comité de Protection des Personnes, Ile de France IV) and patients gave written informed consent to participate.

### Plasma Markers of Immune Activation and T-Cell Activation Measurements

Measurement of immune activation was performed just before cART switch and at least 6 months later. In case of viral replication >50 copies/ml during follow-up, patients were excluded from the analysis. IL-1, IL-6, IL-8, IP-10, MCP-1 and TNF-alpha were measured using ProcartaPlex ImmunoAssays (Life Technologies SAS), while sCD163 and sCD14 (Quantikine ELISA kit, Biotechne) and lipopolysaccharide binding protein (LBP, Enzo Life Sciences) were measured with ELISA kits. T-cell activation markers were quantified *via* flow cytometry.

### Demographic Parameters and Background Measurements

Demographic and main viro-immunological characteristics were recorded.

During follow-up, plasma viral load was quantified 2 months after the switch. Viro-immunological parameters were measured at the end of follow-up, i.e., at least 6 months after treatment simplification, together with immune activation markers.

Viral blips were defined as transient low-level viremia (LLV), between 50 and 500 copies/ml, preceded and followed by suppression, i.e., below the quantification limit of the assay (13). To assess the impact on immune activation, we also checked for very-low-level viremia (VLLV) detected at inclusion or during follow-up. VLLV was defined as viremia <50 copies/ml detected by clinical assays with quantification cut-offs of <50 copies/ml (15). LLV and VLLV were also checked in the last 2 years prior to inclusion, according to patient's files, in order to evaluate the impact of previous residual viremia on immune activation.

During follow-up, clinical events and newly prescribed co-medications were also recorded.

### Statistical Analysis

The main demographic and viro-immunological characteristics of the population are described.

We firstly measured changes from the baseline to the end of follow-up for the entire population. Considering the small sample of the study size and that CD4/CD8 at inclusion and IP-10, MCP-1 and LBP trajectories did not follow a normal distribution (Shapiro-Wilk test of normality), variables were compared using matched Wilcoxon-Mann-Whitney test.

Patients were then stratified into those at low and high risk of immune activation, the latter defined by at least one the following parameters: low CD4 nadir, prior AIDS-defining condition or VLLV during follow-up (16). Differences were measured with Wilcoxon-Mann-Whitney. Statistical analyses were performed using R 4.0.3 software.

## Results

### Population Characteristics

Between April 2019 and May 2020, 20 individuals were included in the study (90% men, mean age 57 years, 25 years since known HIV infection, CD4 cell count at inclusion 666 cells/mm3, CD8 count 766 cells/mm3, CD4/CD8 ratio 0.94, CD4 nadir 326 cells/mm3, 18 years on cART, 15% with prior history of AIDS, 6 cART regimens received). Main comorbid conditions were hypertension (55% of subjects), dyslipidemia (30%), hepatitis C (15%) and diabetes (10%).

Reasons for switching antiretroviral therapy were either reducing the number of pills or limiting potential long-term side effects of antiretrovirals. Mean duration of current antiretroviral regimen at switch was 55.7 months (SD 40.6). In the 2 years prior to inclusion, VLLV was detected at least once in 9 patients and a viral blip in one patient with a viral load of 100 copies/ml 17 months before inclusion. Nine subjects had been receiving a combination of 2 NRTIs and 1 NNRTI, nine had been treated with 2 NRTIs and 1 INSTI, while the remaining two patients had been receiving either 1 NRTI, 1 NNRTI and 1 PI or 1 NRTI, 1 INSTI and 1 NNRTI.

Prescribed dual cART consisted in a combination of Dolutegravir and Rilpivirine for 14 patients, and Dolutegravir and Lamivudine for the remaining 6 patients.

### Follow-Up and Trends in Immune Activation Markers According to Risk Factors for Virological Failure

The mean follow-up period was 6.9 months. Each subject maintained a viral load below 50 copies/ml from the start to the end of follow-up. However, 3/20 individuals had VLLV at inclusion, while at the end of follow-up there were 7/20 subjects with VLLV.

No significant co-medications with potential interference on immune activation were prescribed during follow-up. One individual suffered an ischemic stroke and one a pulmonary embolism, both just before the end of follow-up. No clinical event nor intolerance to treatment occurred among the remaining 18 individuals.

From the inclusion to the end of follow-up, switching from a triple to a dual regimen was associated with IP-10 and sCD14 values decrease (38.3 vs. 22.8 pg/mL, *p* < 0.05, 3.6 vs. 3.0 pg/mL, *p* < 0.05, respectively) and sCD163 increase (371.9 vs. 416.0 ng/mL, *p* = 0.09).

**Figure 1:**
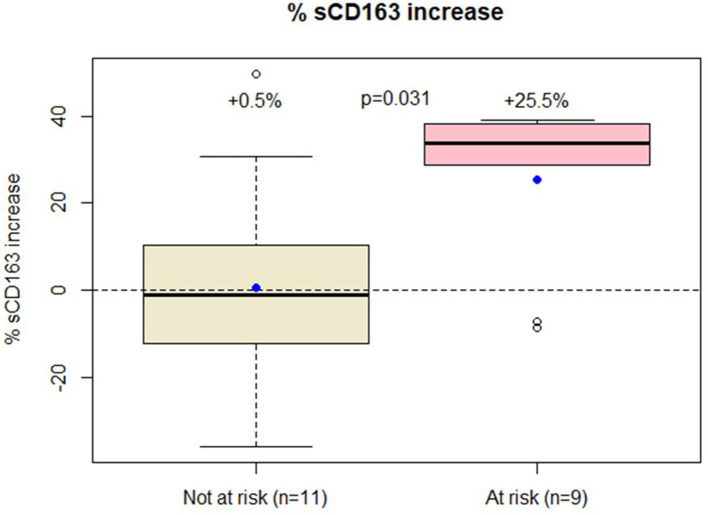
Patients at low risk of immune activation had only modest increase in sCD163 trajectory while those at high risk had over 25% increase in sCD163 values.

A significant increase in sCD163 levels was detected in individuals at risk for immune activation [+ 25.5% (SD = 19.3) vs. +0.5% (SD = 24.6), *p* = 0.02, Figure 1] despite maintaining their viral load <50 cp/ml. No changes were observed for the other immune activation markers (Table 1). No differences in the sCD163 trajectory were found between individuals switching to Dolutegravir and Rilpivirine and those switching to Dolutegravir and Lamivudine (data not shown). Moreover, comorbid conditions were not associated with macrophage activation and sCD163 trajectory did not correlate with the other immune activation markers (data no shown).

**Table 1:** Comparison between subjects at low and high risk of immune activation.

	**At risk-group**		
	**No – *n* = 11**	**Yes – *n* = 9**	
	**Mean [SD]**	**Mean [SD]**	***p*-value^a^**
Age (years)	55.1 [5.5]	58.9 [11.1]	0.503
Years since HIV infection	26.8 [6.3]	22.1 [7.4]	0.119
CD4/CD8 ratio at inclusion	0.9 [0.4]	1.0 [0.4]	0.456
Years on cART	19.0 [5.0]	17.8 [6.6]	0.712
Number of regimens received	6.3 [3.8]	6.0 [2.5]	1.000
CD4/CD8 trajectory (%)	−1.5 [9.8]	5.4 [20.4]	0.657
CD4+CD38+HLADR+ cells trajectory (%)	23.4 [52.4]	86.1 [88.1]	0.256
CD8+CD38+HLADR+ cells trajectory (%)	28.3 [99.7]	346.8 [581.4]	0.130
C-reactive protein trajectory (%)	−8.0 [37.2]	447.6 [1277.0]	0.958
IP 10 trajectory (%)	−35.8 [12.5]	43.1 [247.6]	0.201
MCP 1 trajectory (%)	−11.8 [21.7]	31.9 [153.8]	0.766
sCD14 trajectory (%)	−18.4 [15.7]	−12.7 [9.6]	0.456
sCD163 trajectory (%)	0.5 [24.6]	25.5 [19.3]	**0.031**
LBP trajectory (%)	−11.5 [29.6]	31.1 [76.6]	0.175

* *Wilcoxon-Mann-Whitney test*.

*Group at risk: at least one parameter between low CD4 nadir, previous AIDS stage or very low level viremia during the follow-up*.

*cART, combination antiretroviral treatment; IP 10, Interferon γ protein 10; MCP 1, Monocyte Chemo-attractant Protein-1; LBP, Lipopolysaccharide Binding Protein*.

Besides, neither abacavir- nor tenofovir- containing regimen before the switch were associated with the trajectory of inflammatory markers (data not shown). The prescription of integrase-inhibitor also did not correlate with such markers (data not shown).

In a subgroup of 11 individuals in whom T-cell activation subsets were also available, neither CD4+CD38+HLA-DR+ nor CD8CD38+HLA-DR+ cells differed between the group at low and high risk of immune activation (+ 23.4 vs. + 86.1%, *p* = 0.17, + 28.3 vs. 346.8%, *p* = 0.35, respectively).

## Discussion

In a population of successfully treated individuals living with HIV switching from a triple-drug to a dual-drug regimen as a simplification strategy, we found that sCD163, a well-known marker of macrophage activation (17), increased in patients with a low CD4 nadir, a previous AIDS-defining event or detectable residual viremia during follow-up, despite sustained virological control (18). Interestingly, in line with our previous observation that sCD163 and sCD14 are distinct markers of monocyte activation (19), there was no difference for sCD14 between the two groups we analyzed. However, when comparing changes from baseline to the end of follow-up for the entire population, sCD14 and IP-10 even improved. Reasons for such improvement need to be elucidated. Although the clinical design of our study was different, Gonzalez-Cordon et al. previously observed sCD14 decrease in patients switching from boosted PI to Dolutegravir (20).

To our knowledge, our study investigated the broadest range of inflammatory markers in HIV-infected patients simplifying their treatment.

Dual cART regimens showed their non-inferiority in terms of plasmatic virological control, and data on the genital compartment are also encouraging (21). However, data about the impact of dual therapy on immune activation and systemic inflammation are still scarce. Our results confirm that the majority of inflammatory markers do not change after treatment simplification. However, we found that a significant macrophage activation could be observed. The clinical impact of such monocyte/macrophage activation needs to be elucidated, but sCD163 has been shown as a risk factor for several non-AIDS-defining events, such as HIV-associated neurocognitive disorders, cardiovascular disease or liver fibrosis (22–24). In particular, it probably represents one of the best marker of the atherosclerotic plaque formation in HIV-infected individuals but also in the general population (25). Moreover, as ADAM-17 and TNF-alpha, the major stimuli for sCD163 shedding, are implicated in the replication of quiescent CD4+ T cells, sCD163 could also be associated with residual viremia originating from a reservoir of long lived cells (26). Castley et al. did not find such association with cardiovascular diseases and residual viremia for sCD14, thus confirming that these markers have distinct pathways and potentially explaining the differences in their trajectories we found in our study (26). In other terms, sCD163 seems to offer better information regarding cardiovascular inflammation as well as HIV treatment responses beyond the detection threshold of viral load measurements.

If confirmed by larger and controled studies, our results suggest that switching to a dual-drug regimen could increase the risk of immune activation and thereby of potential non-AIDS events despite virological control. Consequently, in patients with prior severe HIV disease, i.e., AIDS-defining condition and/or low CD4 nadir, treatment should not be simplified in favor of dual-drug regimens, while the occurrence of residual replication after treatment simplification could jeopardize the continuation of such dual-drug therapies.

Our results are in line with those presented by Serrano-Villar et al. who recently showed that in patients switching to dual cART, a delayed increase in 3 plasmatic markers of inflammation was observed (14). However, our study suggests that the effects on macrophage activation could appear much earlier, i.e., during the first year following the switch. Moreover, Belmonti et al. did not find any change in inflammatory markers in patients switching to a dual therapy, but signals of macrophage activation did not include sCD163 (13).

Causes of such immune activation when reducing the number of effective molecules are still unknown. We suggest this might be due to diminished drug pressure on either tissue or cellular reservoirs. Indeed, it has been shown that INSTI diffusion in lymph nodes, with the exception of Elvitegravir, is generally poor (27), thus potentially exposing the virus to sub-optimal concentrations of antiretroviral compounds in this reservoir. Besides, the fact that the percentage of patients with VLLV increased after cART simplification could be in favor of an association between immune activation and poorer pharmacological control in reservoirs.

Unfortunately, we did not measure HIV-DNA nor cellular markers of monocyte/macrophage activation, which could, respectively, improve our understanding of viral replication mechanisms in the cellular reservoir and identify which subsets of peripheral blood mononuclear cells are most activated.

One of our study limitations, aside from the small number of patients, is its single arm design. However, although we did not include a comparative arm with patients continuing a triple-drug regimen, it is very unlikely in our view that in a population of successfully treated subjects on stable therapy and no other clinical events or medical interventions, such differences in macrophage activation could be explained by other factors than treatment simplification.

In conclusion, cART simplification in favor of dual-drug therapy could be associated with increased macrophage activation. The clinical consequences of long-term immune activation are still unknown, but further prospective studies are urgent before generalizing the prescription of dual-drug regimens.

## Data Availability Statement

The original contributions presented in the study are included in the article/supplementary material, further inquiries can be directed to the corresponding author/s.

## Ethics Statement

The studies involving human participants were reviewed and approved by Paris Ethics Committee (Comité de Protection des Personnes, Ile de France IV). The patients/participants provided their written informed consent to participate in this study.

## Author Contributions

MV, JD, RF, and CP designed the study. MV wrote the manuscript. RF and CP performed the statistics and interpreted the data. MT, RC, and PC analyzed immune activation markers. All authors contributed to the article and approved the submitted version.

## Conflict of Interest

The authors declare that the research was conducted in the absence of any commercial or financial relationships that could be construed as a potential conflict of interest.

## Publisher's Note

All claims expressed in this article are solely those of the authors and do not necessarily represent those of their affiliated organizations, or those of the publisher, the editors and the reviewers. Any product that may be evaluated in this article, or claim that may be made by its manufacturer, is not guaranteed or endorsed by the publisher.

## References

[1] Vella S Schwartländer B Sow SP Eholie SP Murphy RL. The history of antiretroviral therapy and of its implementation in resource-limited areas of the world. AIDS. (2012) 26:1231–41. 10.1097/QAD.0b013e32835521a3 22706009

[2] European AIDS Clinical Society Guidelines. (2017). Available online ta: http://www.eacsociety.org/files/guidelines_8.2-english.pdf (accessed March 16, 2021).

[3] World Health Organization Consolidated Guidelines on the Use of Antiretroviral Drugs For Treating and Preventing of HIV Infection. Recomendations for a Public Approach. 2nd ed. (2016). Available online at: http://apps.who.int/iris/bitstream/10665/208825/1/9789241549684_eng.pdf (accessed March 16, 2021).

[4] Serrano-Villar S Pérez-Elías MJ Dronda F Casado JL Moreno A Royuela A. Increased risk of serious non-AIDS-related events in HIV-infected subjects on antiretroviral therapy associated with a low CD4/CD8 ratio. PLoS ONE. (2014) 9:e85798 10.1371/journal.pone.0085798 24497929 PMC3907380

[5] DHHS Guidelines for the Use of Antiretroviral Agents in Adults Adolescents Living with HIV. Available online at: https://aidsinfo.nih.gov/guidelines (accessed March 16, 2021).

[6] Cento V Perno FC. Two-drug regimens with dolutegravir plus rilpivirine or lamivudine in HIV-1 treatment-naïve, virologically-suppressed patients: Latest evidence from the literature on their efficacy and safety. J Global Antimicrob Resist. (2020) 20:228–37 10.1016/j.jgar.2019.08.010 31446092

[7] Achhra AC Boyd MA. Antiretroviral regimens sparing agents from the nucleoside(tide) reverse transcriptase inhibitor class: a review of the recent literature. AIDS Res Ther. (2013) 10:33. 10.1186/1742-6405-10-33 24330617 PMC3874614

[8] Cahn P Rolòn MJ Figueroa MI Gun A Paerson P Sued O. Dolutegravir lamivudine as initial therapy in HIV-infected, ARV naive patients: 48 week results of the PADDLE study. J Int AIDS Soc. (2017) 20:21678. 10.7448/IAS.20.01.21678 28537061 PMC5515053

[9] Cahn P Madero JS Arribas JR Antinori A Ortiz R Clarke AE. Dolutegravir plus lamivudine versus dolutegravir plus tenofovir disoproxil fumarate and emtricitabine in antiretroviral-naive adults with HIV-1 infection (GEMINI-1 and GEMINI-2): week 48 results from two multicentre, double-blind, randomised, non-inferiority, phase 3 trials. Lancet. (2019) 393:143–55 10.1016/S0140-6736(18)32462-0 30420123

[10] Mussini C Lorenzini P Cozzi-Lepri A Marchetti G Rusconi S Gori A. Switching to dual/monotherapy determines an increase in CD8+ in HIV-infected individuals: an observational cohort study. BMC Med. (2018) 16:79. 10.1186/s12916-018-1046-2 29807541 PMC5972434

[11] Caby A Lambert-Niclot S Guiguet M Boutolleau D Agher R Valantin MA. Determinants of a low CD4/CD8 ratio in HIV-1-infected individuals despite long-term viral suppression. Clin Infect Dis. (2016) 62:1297–303. 10.1093/cid/ciw076 26908792

[12] Riddler SA Aga E Bosch RJ Bastow B Bedison M Vagratian D. Continued slow decay of the residual plasma viremia level in HIV-1-infected adults receiving long-term antiretroviral therapy. J Infect Dis. (2016) 213:556–60 10.1093/infdis/jiv433 26333941 PMC4721905

[13] Belmonti S Lombardi F Quiros-Roldan E Latini A Castagna A Borghetti A. Systemic inflammation markers after simplification to atazanavir/ritonavir plus lamivudine in virologically suppressed HIV-1-infected patients: ATLAS-M substudy. Antimicrob Chemother. (2018) 73:1949–54. 10.1093/jac/dky125 29788156

[14] Serrano Villar S Lopez-Huertas MR Gutierrez F Beltran M Pons M Viciana P. Reducing ART to Less Than 3-ARV Regimen Linked to Increased Systemic Inflammation. Stockholm: OAB0304 International AIDS Society (IAS) Congress (2020).

[15] Ryscavage P Kelly S Z Li J Harrigan PR Taiwo B. Significance and clinical management of persistent low-level viremia and very-low-level viremia in HIV-1-infected patients. Antimicrob Agents Chemotherapy. (2014) 58:3585–98 10.1128/AAC.00076-14 24733471 PMC4068602

[16] Lambert-Niclot S Flandre P Valantin MA Peytavin G Duvivier C Haim-Boukobza S. Factors associated with virological failure in HIV-1-infected patients receiving darunavir/ritonavir monotherapy. J Infect Dis. (2011) 204:1211–6. 10.1093/infdis/jir518 21917894

[17] Wallet MA Rodriguez CA Yin L Saporta S Chinratanapisit S Hou W. Microbial translocation induces persistent macrophage activation unrelated to HIV-1 levels or T-cell activation following therapy. AIDS. (2010) 24:1281–90. 10.1097/QAD.0b013e328339e228 20559035 PMC2888494

[18] Hernandez B Kahl L Matthews J Vincent T Angelis K Koteff J. Bone, Renal and Inflammatory Biomarkers up to Week 100 Post Switch to DTG + RPV: the SWORD-1 and SWORD-2 Studies. HIV & Hepatitis Nordic Stockholm, Sweden, poster P9 (2018).

[19] Psomas C Younas M Reynes C Cezar R Portalès P Tuaillon E. One of the immune activation profiles observed in HIV-1-infected adults with suppressed viremia is linked to metabolic syndrome: the ACTIVIH study. EbioMedicine. (2016) 8:265–76 10.1016/j.ebiom.2016.05.008 27428436 PMC4919610

[20] Gonzalez-Cordon A Assoumou L Moyle G Waters L Johnson M Domingo P. Switching from boosted PIs to Dolutegravir decreases soluble CD14 adiponectine in high cardiovascular risk people living with HIV. J Antimicrob Chemother. (2021). 10.1093/jac/dkab158. [Epub ahead of print] 34120186

[21] Charpentier C Peytavin G Raffi F Burdet C Landman R Lê M. Pharmacovirological analyses of blood and male genital compartment in patients receiving dolutegravir + lamivudine dual therapy as a switch strategy (ANRS 167 LAMIDOL trial). J Antimicrob Chemother. (2020) 75:1611–7. 10.1093/jac/dkaa035 32091102

[22] Yadav A Kossenkov AV Knecht VR Showe LC Ratcliffe SJ Montaner LJ. Evidence for persistent monocyte and immune dysregulation after prolonged viral suppression despite normalization of monocyte subsets, sCD14 and sCD163 in HIV-infected individuals. Pathog Immun. (2019) 4:324–62. 10.20411/pai.v4i2.336 31893252 PMC6930814

[23] Lidofsky A Holmes JA Feeney ER Kruger AJ Salloum S Zheng. Macrophage activation marker soluble CD163 is a dynamic marker of liver fibrogenesis in human immunodeficiency virus/hepatitis c virus coinfection. J Infect Dis. (2018) 218:1394–403. 10.1093/infdis/jiy331 29868909 PMC6151081

[24] Sherman KE Meeds H. .L. Rouster S.D. Abdel-Hameed EA Hernandez J Tamargo J. Soluble CD163 identifies those at risk for increased hepatic inflammation & fibrosis. Open Forum Infect Dis. (2021) 8:ofab203. 10.1093/ofid/ofab203 34104667 PMC8180248

[25] McKibben RA Margolick JB Grinspoon S Li X Palella Jr FJ. Elevated levels of monocyte activation markers are associated with subclinical atherosclerosis in men with and those without HIV infection. J Infect Dis. (2015) 211:1219–28. 10.1093/infdis/jiu594 25362192 PMC4402336

[26] Castley A Williams L James I Guelfi G Berry C Nolan D. Plasma CXCL10, sCD163 and sCD14 levels have distinct associations with antiretroviral treatment and cardiovascular disease risk factors. PLoS ONE. (2016) 11:e0158169. 10.1371/journal.pone.0158169 27355513 PMC4927121

[27] Dyavar SR Gautam N Podany AT Winchester LC Weinhold JA Mykris TM. Assessing the lymphoid tissue bioavailability of antiretrovirals in human primary lymphoid endothelial cells and in mice. J Antimicrob Chemother. (2019) 74:2974–8. 10.1093/jac/dkz273 31335938 PMC6753470

